# On the Application of Dialysis in Determining the Nature of the Crystaline Constituents of Plants

**Published:** 1864-12

**Authors:** J. Attfield


On the Application of Dialysis in Determining the Nature of the Crystaline Constituents of Plants.—By J. Attfield, ph. d., f. c. s.—The author had dialysed a few plant-juices, the first that came to hand, and from each had obtained some of the crystaline constituents. The tops of the common potato yielded a crop of nitrate of potash, some cubes of chloride of potassium, hexagonal crystal not analysed, sugar, and an amonia salt. The deadly night-shade gave nitrate of potash, an unknown magnesia salt in square prisms, sugar, etc. Pea-pole yielded only sugar. The common garden lettuce contained nitrate of potash, tetrahedra of undetermined composition, sugar and ammonia. Cucumbers furnished sugar, ammonia and sulphate of lime. The cabbage also furnished sulphate of lime and ammonia. Stramonium contained so much nitrate of potash, that dried portions quite deflagrated on being ignited.

From these experiments the author thought the proposed application of dialosis promised to be of great service, directly and indirectly, in investigating vegetable physiology.—*Journal of Pharmacy.*

